# Effect of Curing Mode on Shear Bond Strength of Self-Adhesive Cement to Composite Blocks

**DOI:** 10.3390/ma9030210

**Published:** 2016-03-18

**Authors:** Jin-Young Kim, Ga-Young Cho, Byoung-Duck Roh, Yooseok Shin

**Affiliations:** 1 Department of Conservative Dentistry, College of Medicine, Inha University, Seoul 03722, Korea; jjin02k@gmail.com (J.-Y.K.); ecobleu7@gmail.com (G.-Y.C.); 2 Department of Conservative Dentistry, Oral science research center and microscope center, College of Dentistry, Yonsei University, 50-1 Yonsei-ro, Seodaemun-Gu, Seoul 03722, Korea; operatys16@yuhs.ac

**Keywords:** resin nano ceramic, self-adhesive resin cement, curing mode, fracture pattern

## Abstract

To overcome the disadvantages of computer-aided design/computer-aided manufacturing (CAD/CAM) processed indirect restorations using glass-ceramics and other ceramics, resin nano ceramic, which has high strength and wear resistance with improved polish retention and optical properties, was introduced. The purpose of this study was to evaluate the shear bond strength and fracture pattern of indirect CAD/CAM composite blocks cemented with two self-etch adhesive cements with different curing modes. Sand-blasted CAD/CAM composite blocks were cemented using conventional resin cement, Rely X Ultimate Clicker (RXC, 3M ESPE, St. Paul, MN, USA) with Single Bond Universal (SB, 3M ESPE, St. Paul, MN, USA) for the control group or two self-adhesive resin cements: Rely X U200 (RXU, 3M ESPE, St. Paul, MN, USA) and G-CEM Cerasmart (GC, GC corporation, Tokyo, Japan). RXU and GC groups included different curing modes (light-curing (*L*) and auto-curing (*A*)). Shear bond strength (SBS) analyses were performed on all the specimens. The RXC group revealed the highest SBS and the GC *A* group revealed the lowest SBS. According to Tukey’s post hoc test, the RXC group showed a significant difference compared to the GC *A* group (*p* < 0.05). For the curing mode, RXU *A* and RXU *L* did not show any significant difference between groups and GC *A* and GC *L* did not show any significant difference either. Most of the groups except RXC and RXU *L* revealed adhesive failure patterns predominantly. The RXC group showed a predominant cohesive failure pattern in their CAD/CAM composite, Lava^TM^ Ultimate (LU, 3M ESPE, St. Paul, MN, USA). Within the limitations of this study, no significant difference was found regarding curing modes but more mixed fracture patterns were showed when using the light-curing mode than when using the self-curing mode.

## 1. Introduction

With the development of CAD/CAM techniques, dental restorations can be made with one appointment. There are two main types of materials currently available for esthetic CAD/CAM indirect restorations: glass-ceramics/ceramics and resin composites. Ceramic CAD/CAM indirect restorations have some disadvantages and limitations, such as friability and higher opposite enamel wear [1]. For compensating the disadvantages of ceramic CAD/CAM indirect restorations, resin nano ceramic (Lava^TM^ Ultimate, 3M ESPE, St. Paul, MN, USA) was introduced for CAD/CAM restoration materials. This nano resin ceramic material is based on the combination of nanotechnology and ceramic [2]. It contains a blend of three fillers, silica and zirconia nanoclusters and individual nanoparticles, with a total filler load of approximately 80 wt. %. It is intended to offer the ease of handling of a composite material with a surface gloss and finish retention similar to porcelain. The specific selection of the material contents of the dental restoration as well as the bonding and cementing system affect the adhesion properties [2].

Self-adhesive dual cured resin cements were introduced to simplify the step of the bonding procedure and to overcome the limitations of complex multistep applications, susceptibility to moisture and possible postoperative sensitivity of conventional resin cements [3,4]. Acidic monomers in self-adhesive resin cements demineralize and infiltrate the tooth substrate, providing micromechanical retention [5]. Simultaneously, the reaction between the phosphoric acid monomers of the cements and hydroxyapatite of the tooth substrate can offer chemical retention. However, many studies have reported the poor adhesion to dentin and the low bond strength as flaws of self-adhesive resin cements [4,6,7]. Although self-adhesive resin cements reduced the complex multisteps and postoperative sensitivity, the limited etching potential and superficial interaction with the dentin surface provided a lower bond strength than conventional resin cements [5]. Moreover, cement itself is too viscous to penetrate into the demineralized collagen fiber network.

Most resin cements are dual-cured, containing both self-cured and light-cured components [8]. In most clinical cases, dental cement is difficult to cure only with the light-cure mode because of the distance from the light source due to the thickness and opacity of the restoration [8,9]. Several studies demonstrated that self-curing alone is not as effective as light activation in dual-cured resin cements when evaluating the degree of conversion, cement hardness, rate of polymerization, solubility, and bond strength [8,10]. The low degree of the conversion rate of the self-curing mode can be influenced by the increase of water sorption and solubility, which may compromise the mechanical properties of the resin cements and the longevity of indirect restoration [11,12].

Adequate surface activation and the increasing roughness of indirect, polymerized composite resins through various surface treatments provide a better mechanical interlocking and a stronger chemical bond to the cement [9]. According to the manufacturer’s recommendations, the Lava^TM^ Ultimate (LU) restorative does not require HF etching and sandblasting with alumina oxide is recommended. Depending on the cement, the LU restorative might need to be pretreated with a silane and/or a bonding agent. Silane is a bifunctional molecule that bonds through siloxane to the exposed fillers in the composite [13] and can increase the bond strength by improving the wettability of the treated surface. Before silanization, through the sandblasting with alumina oxide, the resin matrix of the surface is partially destroyed and the filler particles are exposed [14]. This leads to better mechanical interlocking between the restoration and the substructure. Independent studies about bond strength and preferred treatment protocols for this increasingly popular material group are needed.

In this study, we measured the shear bond strength of three types of resin cement to indirect CAD/CAM composite blocks using different curing modes and aimed to evaluate the effect of curing mode on self-adhesive resin cements to indirect CAD/CAM composite blocks. The hypothesis tested were these: (1) no differences in shear bond strength exist between two self-adhesive resin cements; (2) the curing modes affect the shear bond strength.

## 2. Materials and Methods

### 2.1. Pre-Treatment of CAD/CAM Composite

The CAD/CAM composite blocks (Lava^TM^ Ultimate 3M ESPE, St. Paul, MN, USA) were sectioned into three smaller blocks (thickness of 3 mm) using a low-speed precision diamond saw (Metsaw-LS, TOPMET, Daejeon, Korea) under water irrigation. Afterwards, their surfaces were sectioned into nine parts with the same dimensions using a low-speed diamond disk. Blocks were mounted in ring plastic holders made of self-cured acrylic resin. The uppermost surface was sanded using 600 grit sandpaper and then sandblasted with aluminum oxide (50 um) for 60 s at 0.3 MPa air pressure at a direction perpendicular to the surface and cleaned with distilled water for 60 s in ultrasonic bath.

### 2.2. Cementation and Curing Modes

**Figure 1:**
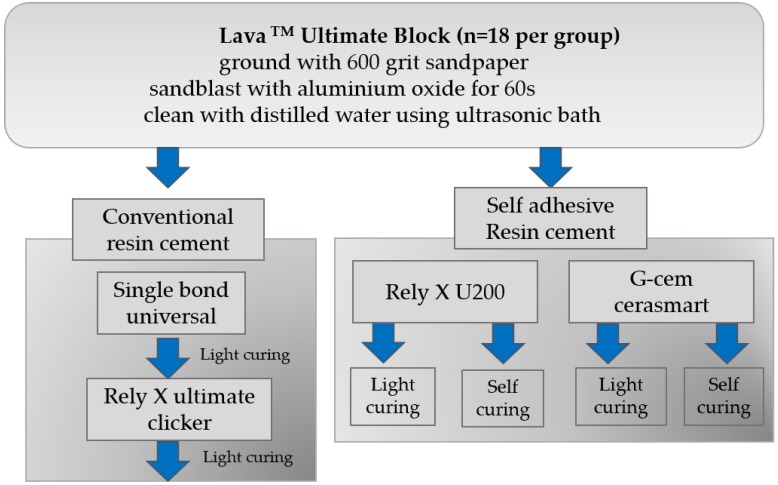
Flowchart of the cementation procedure on the specimens.

Sand-blasted CAD/CAM composite blocks (Lava^TM^ Ultimate, LU, 3M ESPE) were cemented using the conventional resin cement Rely X Ultimate Clicker (RXC, 3M ESPE) with Single Bond Universal (SB, 3M ESPE) for control group or two self-adhesive resin cements: Rely X U200 (RXU, 3M ESPE) and G-CEM Cerasmart (GC, GC Corporation, Tokyo, Japan). For RXC group, Single Bond Universal was applied in one coat for 20 s application time and air dried for 5 s and light-cured for 10 s with 1200 mW LED light-curing unit (DB-686 Cappu LED curing light; Bisco Asia, Seoul, Korea). Light-curing unit intensity of light was measured before experiment. For an application of the cement on the sandblasted CAD/CAM composite block, the tube of the same dimension was fixed on the block with wax and the cement was mixed according to the manufacturer’s recommendation and put into the tube and light-cured for 20 s. (Table 1) RXU and GC groups included different curing modes (light-curing mode (*L**)*** and auto-curing mode (*A*)). In light-curing group, the cement was light-cured for 20 s with 1200 mW LED light-curing unit, while the *A* groups were left for 1 h at room temperature in darkness to auto-cure. Five groups included 18 specimens each. Before testing, all the specimens were kept in a humid environment at room temperature for 24 h after cementation. The procedure of specimen preparation is illustrated in Figure 1.

### 2.3. Preparation for Micro-Shear Bond Strength Testing

Specimens were mounted on the jig of a universal testing machine (Bisco, Schaumburg, IL, USA) and shear force at a crosshead speed of 0.5 mm/min was applied to the adhesive interface until fracture occurred. The force (N) at which the bond failed was recorded and divided into 4.45 mm^2^, the contact area of resin cement and the block. The bond strength was calculated in MPa. Data were analyzed using one way analysis of variance (ANOVA) with Tukey’s post hoc test. After testing, the failure mode was determined with digital camera with magnifications to classify the type of fracture and was recorded as “cohesive failure in resin cement” and “adhesive failure at the interface cement-CAD/CAM composite” and “mixed failure”. Selected specimens from each characterized fracture pattern were examined by scanning electron microscopy (SEM).

## 3. Results

### 3.1. SBS

The mean SBS and descriptive statistics according to different cements and curing modes are mentioned in Table 2. The RXC group revealed the highest SBS and the GC *A* group revealed the lowest SBS. According to Tukey’s post hoc test, the RXC group showed a significant difference compared to the GC *A* group (*p* < 0.05) and the RXU *A* group showed a significant difference compared to the GC *A* group (*p* < 0.05). For the curing mode, RXU *A* and RXU *L* did not show any significant difference between the groups and GC *A* and GC *L* did not show any significant difference either.

### 3.2. Failure Mode Analysis Using a Scanning Electron Microscope (SEM)

**Figure 2:**
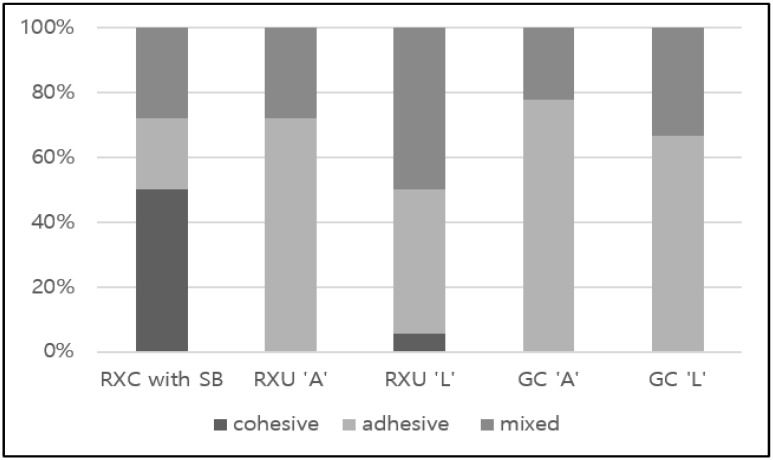
Percentage of failure modes of the tested groups.

**Figure 3:**
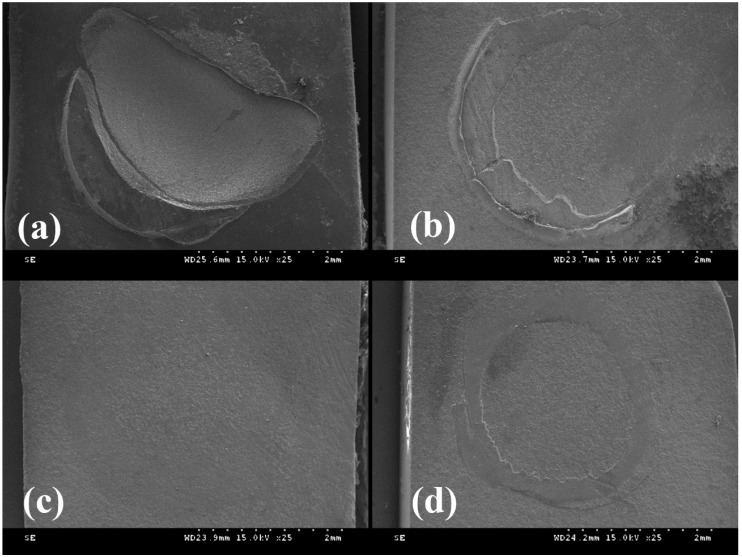
SEM photomicrographs of fractured surfaces: (**a**) Cohesive fracture pattern showed on RXU group with SB; (**b**,**d**) Mixed fracture pattern showed on RXU *L* and GC *L* group; (**c**) Adhesive fracture pattern showed on GC *A* group.

The failure pattern distribution (%) of each group is shown in Figure 2. Most of the groups except RXC and RXU *L* revealed adhesive failure patterns along the resin cement-CAD/CAM resin block interface predominantly. Figure 3 shows the representative SEM micrographs of the fractured block sides. The RXC group showed a predominant cohesive failure mode in its CAD/CAM composite failure (Figure 3a). In a specimen of the RXU *L* and GC *L* groups, an adhesive failure region along the block-cement interface revealed a remnant of resin cements (Figure 3b,d). In a specimen of the GC *A* group, complete separation of the luting cement from the CAD/CAM composite blocks was shown (Figure 3c).

## 4. Discussion

Ceramic materials have some important properties, such as translucency, chemical stability, fluorescence, biocompatibility, a high resistance to compression, and a coefficient of thermal expansion similar to tooth structure, but they also have some disadvantages and limitations, such as friability and higher opposite enamel wear, and susceptibility to fracture propagation, which may cause complications in dynamic occlusion over time [1]. Part of a new category of CAD/CAM materials, resin nano ceramic, Lava™ Ultimate consists of 80 wt. % nano ceramic and 20 wt. % resin. The 80 wt. % nano ceramic was composed of 4–11 nm zirconia and 20 nm silica particles, which are clustered and surface-treated by nanotechnology. The 20 wt. % resin is a highly cross-linked polymer processed in a proprietary high temperature for multiple hours.

According to the manufacturer’s recommendation, the LU restorative might need to be pretreated with a silane and/or a bonding agent depending on the cement. The required cement is an adhesive resin cement or self-adhesive resin cement. The retention of ceramic restorations can be achieved by chemical etching with acidic fluorides and subsequent treatment of a silane coupling agent prior to bonding with resin cement. However, LU restorative does not require HF etching because resin nano ceramic is not etchable. Sandblasting with alumina oxide, with a grain size of 50 microns or less, at 2 bar pressure is recommended [15].

In this study, specimens cemented with Rely X Ultimate Clicker after application of Single Bond Universal and light-cured exhibited the highest shear bond strength. Single Bond Universal adhesive has the ability to bond to indirect substrates without the use of separate priming agents. Single Bond Universal contains various components such as silane, MDP phosphate monomer, Vitrebond™ copolymer, *etc.* Silane can function as a primer for etched glass surfaces and the silica component of resin nano ceramics. The acidity of the primer probably promoted the formation of the siloxane bonds between the silane coupling agent and ceramic surface, and contributed to the high bond strength values [16,17]. This acidic primer may also be attributable to increased wetting at the interface, thus promoting the condensation of silane to the composite [18]. MDP phosphate monomer is a proven self-etch monomer and primer for zirconia, alumina, metals and zirconia components of resin nano ceramic [19]. When phosphate monomer is applied to zirconia, the hydrogen group of the phosphate monomer and the oxygen group of zirconia slowly react to produce water molecules and to form a stable Zr-O-P covalent bond [20]. The other component, Vitrebond™ copolymer, allows bonding to moist or dry dentin in the total-etch technique, but in this study, the bonding strength of the resin cements to the resin nano ceramics was measured. Therefore, the ability of Vitrebond™ copolymer which can bond to dentin might be ignored.

Components of conventional resin cements are similar to that of composite resin and show superior physical properties to other adhesive-based resin cements. Conventional resin cements might exhibit higher durability and bonding strength when used with adequate dentin bonding agent. In many studies, conventional resin cements exhibited superior bonding strength to other self-adhesive resin cements [21,22]. In this study, the group cemented with the conventional resin cement exhibited the highest bonding strength, but only showed a significant difference with the GC auto-curing group. When comparing the shear bond strength of two types of self-adhesive resin cements in this study, RXU exhibited significantly higher SBS than GC when self-cured. The difference between filler content can be one explanation [23]. However, no significant difference was noted between the shear bond strength of two self-adhesive resin cements cured by the light-curing mode.

Many studies evaluating hardness, flexural strength and the degree of conversion of dual-cured resin cements have shown that self-curing components of most resin cements were not enough to compensate for the attenuation in light intensity or total absence of light and the self-curing mode of resin cements is less effective when compared to the dual-cured or photo-cured cements [8,9,10,24,25,26,27,28]. The changes in viscosity during the auto-polymerization reaction reduced the ability of radicals to migrate and continue the conversion reaction [29]. Moreover, the slow polymerization promoted by the self-curing mode might have been impaired by water produced during the neutralization reaction of the phosphate monomers with basic filler within the self-adhesive resin cement. In this study, without light activation, the shear bond strength of G-CEM Cerasmart was lower than that of the light-cured group but no significant differences (*p* > 0.05) were noted. Without light activation, the shear bond strength of Rely X U200 was slightly higher than that of the light-cured group but no significant differences were noted (*p* > 0.05).

Benzoyl peroxide, which is a representative initiator component for self-curing, can initiate the polymerization process by oxidation-reduction reaction with the tertiary amine [30]. However, acid in adhesive can react with the tertiary amine and lead to the formation of quaternary ammonium and diminish the polymerization process [30]. The RXU *A* group exhibited similar shear bond strength to the RXU *L* group. Rely X U200 includes sodium *p*-toluen sulfonate as an initiator. Suh *et al.* [31] reported that an initiator such as sodium aryl sulfate or aryl-borate salt will be needed to prevent the reaction of the acidic monomer with the tertiary amine which is an initiator for chemical polymerization. Sodium salt can initiate the polymerization process by a reaction with the acidic resin monomer and formation of phenyl or the benzenesulfonyl free radical. Shear bond strength of groups cemented with Rely X U 200 can be lowered because of higher viscosity from higher filler content at an early age, but might be increased through additional acid-base reaction [32,33].

The GC *L* group showed higher shear bond strength than the GC *A* group but no significant difference was noted. According to the manufacturer of the G-CEM Cerasmart, they used a new innovative chemical initiator system offering the highest polymerization in the self-curing mode. They claimed that it polymerizes within four minutes in self-curing mode and provides the maximum bond strength after 20 min.

Toledano *et al.* reported that cohesive and mixed failure patterns are desirable in a clinical situation when analyzing the fracture patterns of specimens [34]. Fischer *et al.* reported that bonding strength can be influenced by the internal strength of the cements and the strength between the adhered materials and cements [35]. According to Asmussen *et al.*, the physical property of the resin cements can be influenced by the type of monomer, filler content, structure, and degree of conversion [36]. Most of the groups except RXC with SB and RXU *L* revealed adhesive failure patterns along the resin cement-CAD/CAM resin block interface. The RXC group showed a predominant cohesive failure pattern in its CAD/CAM composite (50%) failure and these are indicative of more efficient interfacial polymerization. When comparing RXU *A* with RXU *L* and GC *A* with GC *L*, a higher percentage of mixed failure was observed in the RXU *L* and GC *L* groups. In microscopic observation through SEM, the RXC group showed a predominant cohesive failure pattern in its CAD/CAM composite failure. In a specimen from the RXU *L* and GC *L* groups, an adhesive failure along the block-cement interface revealed the remnants of resin cements. In a specimen of the GC *A* group, complete separation of the luting cement from the CAD/CAM composite blocks was shown.

Since this study was conducted to evaluate the specific interaction between each cement type and LU restoration, a simple design for attaching the cement to the CAD/CAM resin block was used. To obtain more realistic results in clinical cases, the bond strength may need to be measured after the teeth and the restoration are bonded, after which the fracture surface may need to be examined. Parameters such as restoration thickness and cement thickness determine the attenuation of light [37,38]. Therefore, it is needed to consider other factors that affect the curing mode. Further comparative studies on additional physical properties such as tensile strength, flexure strength, wettability, and color change may be needed in the future.

## Figures and Tables

**Table 1:** Composition and information of dentin adhesive systems and rewetting agents used in this study.

Materials	Code	Material	Batch No.	Compositions	Manufactures
**CAD/CAM Block**	-	Lava^TM^ Ultimate	-	Cured dental restorative, consisting of silica nanomers (20 nm), zirconia nanomers (4–11 nm), nanocluster particles derived from the nanomers (0.6–1.0 um), silane coupling agent, resin matrix	3M, ESPE
**Adhesive**	SB	Single Bond Universal	Lot 553077	MDP Phosphate monomer, dimethacrylate resins, HEMA, Vitrebond™ copolymer, filler, ethanol, water, initiators, silane	3M, ESPE
**Conventional Resin Cement**	RXC	Rely X Ultimate Clicker	Lot 589109	Base paste: methacrylate monomers, radiopaque, silanated fillers, initiator components, stabilizers, rheological additives Catalyst paste: methacrylate monomers, radiopaque alkaline (basic) fillers, initiator components, stabilizers, pigments, rheological additives, fluorescence dye, dual-cure activator for single bond universal adhesive	3M, ESPE
**Self-Adhesive Resin Cement**	RXU	Rely X U200	Lot 590559	Base paste: methacrylate monomers containing phosphoric acid groups, methacrylate monomers, silanated fillers, initiator components, stabilizers, rheological additives Catalyst paste: methacrylate monomers, alkaline (basic) fillers, silanated fillers, initiator components, stabilizers, pigments, rheological additives	3M, ESPE
**-**	GC	G-CEM Cerasmart	Lot 1501061	Paste A: fluoro-alumino-silicate glass, UDMA, dimethacrylate, silicon dioxide, initiator, inhibitor Paste B: silicon dioxide, UDMA, dimethacrylate, initiator, inhibitor	GC corporation

HEMA: 2-hydroxyethyl methacrylate; MDP: methacryloyloxydecyl dihydrogen phosphate; UDMA: urethane dimethacrylate.

**Table 2:** Mean shear bond strength (MPa) and descriptive statistics according to different cements and curing mode.

Group	Curing Mode	N	SBS (SD) (Mpa)
RXC	*L*	18	10.7 ± 5.6 ^a^
RXU	*A*	18	8.9 ± 4.9 ^a,b^
RXU	*L*	18	7.5 ± 4.3 ^a,b,c^
GC	*A*	18	4.5 ± 2.8 ^c^
GC	*L*	18	7.9 ± 4.5 ^a,b,c^
Total	-	90	-

Different superscript letters in the row indicate statistically significant difference (*p* < 0.05); SBS: shear bond strength, SD: standard deviation, L: light-curing, A: auto-curing.
